# Comparing Surgical, Acupuncture, and Exercise Interventions for Improving the Quality of Life in Women With Endometriosis: A Systematic Review

**DOI:** 10.7759/cureus.65257

**Published:** 2024-07-24

**Authors:** Sumayya Afreen, Arvin Perthiani, Elizabeth Sangster, Nidhi Lanka, Prakash Acharya, Shikha Virani, Iana Malasevskaia

**Affiliations:** 1 Department of Obstetrics and Gynecology, California Institute of Behavioral Neurosciences & Psychology, Fairfield, USA; 2 Department of Internal Medicine, Deccan College of Medical Sciences, Hyderabad, IND; 3 Department of General Surgery, California Institute of Behavioral Neurosciences & Psychology, Fairfield, USA; 4 Department of General Surgery, Our Lady of Lourdes Hospital, Drogheda, Louth, IRL; 5 Department of Psychiatry and Behavioral Sciences, California Institute of Behavioral Neurosciences & Psychology, Fairfield, USA; 6 School of Medicine, St. George's University, St. George, GRD; 7 Department of General Medicine, California Institute of Behavioral Neurosciences & Psychology, Fairfield, USA; 8 Department of Internal Medicine, California Institute of Behavioral Neurosciences & Psychology, Fairfield, USA; 9 Department of Medicine, California Institute of Behavioral Neurosciences & Psychology, Fairfield, USA; 10 Department of Medicine, Surat Municipal Institute of Medical Education and Research (SMIMER) Medical College, Surat, IND; 11 Department of Obstetrics and Gynecology, Private Clinic "Yana Alexandr", Sana'a, YEM

**Keywords:** systematic review, exercise, acupuncture, surgical interventions, quality of life, endometriosis

## Abstract

Endometriosis is a chronic inflammatory condition that significantly impacts the quality of life (QoL) of affected women. This systematic review aimed to compare the effects of surgical interventions, acupuncture, and exercise programs on the QoL in women with endometriosis. A comprehensive search was conducted in databases such as PubMed, Cochrane Central Register of Controlled Trials (CENTRAL), Google Scholar, ClinicalTrials.gov, and the World Health Organization International Clinical Trials Registry Platform (WHO ICTRP). Randomized controlled trials (RCTs) and observational studies evaluating the impact of these interventions on the QoL were included. The review identified 10 studies (six RCTs and four observational studies) involving a total of 493 participants. Surgical interventions, particularly the laparoscopic excision of endometriotic lesions, demonstrated substantial reductions in pain and improvements in the QoL. Acupuncture effectively alleviated pain and enhanced overall well-being. Exercise programs improved the QoL, physical function, and pain reduction. The findings suggest that surgical-, acupuncture-, and exercise-based approaches can significantly improve the QoL for women with endometriosis. However, more personalized treatment approaches and further research are needed to understand the long-term benefits, optimal protocols, and underlying mechanisms of these interventions.

## Introduction and background

Endometriosis is a chronic inflammatory disease characterized by the presence of tissue resembling the endometrium outside the uterus, which results in pelvic pain, painful menstruation, and infertility [1]. The causes of endometriosis are multifaceted, involving genetic, immunological, and hormonal factors [2]. This condition severely impacts the quality of life (QoL) due to chronic pain and the psychological burden associated with infertility and persistent symptoms [3,4]. Treatment options commonly include pharmacological methods such as hormonal therapies, surgical procedures to remove or ablate endometriotic lesions, and complementary therapies such as exercise and acupuncture aimed at alleviating symptoms and enhancing overall well-being [5-7].

Approximately 10% of women of reproductive age globally, amounting to around 190 million women, are affected by endometriosis [8,9]. In the United States, the prevalence is approximately one in nine women [10]. Despite its widespread occurrence, the diagnosis of endometriosis often takes an average of seven to 10 years due to nonspecific symptoms and a lack of awareness among healthcare providers [11-13].

While various treatment modalities are available, there is no definitive cure for endometriosis, and the effectiveness of different interventions in improving the QoL remains uncertain [14]. The comparative effectiveness of exercise, acupuncture, and surgery in enhancing the QoL for women with endometriosis has not been thoroughly reviewed. Additionally, the long-term benefits and potential adverse effects of these treatments require further investigation [15]. Understanding the mechanisms through which these interventions alleviate symptoms and improve the QoL is also necessary [16].

This systematic review aims to evaluate the effects of surgical interventions, acupuncture, exercise programs, or a combination of these approaches on the quality of life in women with endometriosis using evidence from randomized controlled trials (RCTs) and observational studies.

## Review

Methods

This systematic review was conducted from May 9, 2024, to June 24, 2024, and it followed the Preferred Reporting Items for Systematic Reviews and Meta-Analyses (PRISMA) guidelines [17]. Data extraction from databases occurred between May 10 and 20, 2024.

Search Strategy

We employed a comprehensive search strategy across five key databases: PubMed/Medical Literature Analysis and Retrieval System Online (MEDLINE), Cochrane Central Register of Controlled Trials (CENTRAL), Google Scholar Advanced Search, ClinicalTrials.gov, and the World Health Organization International Clinical Trials Registry Platform (WHO ICTRP). In PubMed/MEDLINE, we utilized Medical Subject Headings (MeSH) terms related to the population (women with endometriosis), interventions (exercise, acupuncture, and surgery), and outcomes (quality of life and related symptoms). For other databases, including CENTRAL and Google Scholar, we combined relevant keywords with Boolean operators (AND and OR) to refine the search results. This approach yielded a total of 287 studies, with 184 identified from databases and 103 from registers. A detailed breakdown of the search strategy can be found in Table 1.

**Table 1:** Search strategy MEDLINE, Medical Literature Analysis and Retrieval System Online; MeSH, Medical Subject Headings; WHO, World Health Organization

Search strategy	Databases/registers	Filters	Number of studies identified after filters
Endometriosis AND "Quality of life" AND [exercise OR "acupuncture therapy" OR laparoscopy OR "surgical procedure" OR "minimally invasive surgical procedure" OR "gynecologic surgical procedure"]	Cochrane Central Register of Controlled Trials (CENTRAL)	No filters used	86
((("Acupuncture therapy"[MeSH] "Pharmacoacupuncture Treatment"[MeSH] OR "Treatment, Pharmacoacupuncture" OR "Pharmacoacupuncture Therapy" OR "Therapy, Pharmacoacupuncture" OR "Treatment, Acupuncture" OR "Acupuncture Treatments" OR "Acupuncture Treatment" OR "Therapy, Acupuncture" OR "Acupotomies" OR "Acupotomy") OR ("Exercise"[MeSH] OR Exercise*OR "Physical Exercise*" OR "Physical Activit*" OR "Isometric Exercise*") OR ("Surgical Procedures, Operative" OR Laparoscopy OR "Laparoscopic Surger*" OR "Robotic Surgical Procedure*" OR "Gynecologic Surgical Procedure*"[MeSH] OR "Minimally Invasive Surgical Procedure*"[MeSH] OR "Robotic Surgical Procedure*"[Mesh] OR "Laparoscopy"[Mesh]))) AND ("Quality of Life"[MeSH] OR "QOL" OR "Life Quality" OR "Health Related Quality Of Life" OR "Health-Related Quality Of Life" OR "HRQOL") AND ("Endometriosis"[MeSH] OR "Endometriom*" OR "Endometrios*")	PubMed/MEDLINE	Full text, clinical study, clinical trial, observational study, randomized controlled trial, humans, and English	58
allintitle: (endometriosis) AND (QOL OR "quality of life" OR QoL) AND (exercise OR acupuncture OR Surgery OR "surgical") -review -OR -meta-analysis -OR -commentary -OR -editorial -OR -case -report	Google Scholar	Advanced search, all in title, and excluding review -OR -meta-analysis -OR -commentary -OR -editorial -OR -case -report	40
Condition-Endometriosis Outcome- quality of life Intervention - surgery OR exercise OR acupuncture	ClinicalTrials.gov	Female	87
Endometriosis AND quality of life AND (surgery OR exercise OR acupuncture)	WHO International Clinical Trials Registry Platform (ICTRP)	No filters used	16

Study Selection

Once the articles were collected and imported into the EndNote 21 application (Clarivate, Philadelphia, PA), the authors conducted a preliminary screening of titles and abstracts to determine which articles met the criteria for full-text evaluation. This selection process utilized pre-defined inclusion and exclusion criteria. The details of these criteria are outlined in Table 2.

**Table 2:** Inclusion and exclusion criteria for study selection

Criteria	Inclusion	Exclusion
Study	Randomized controlled trials (RCTs) and observational studies	Case reports, reviews, and editorials
Population	Women diagnosed with endometriosis	Nonhuman studies
Intervention	Exercise, acupuncture, and surgical treatments	Interventions not specified or irrelevant to the research question
Outcomes	Improvement in the quality of life (QoL), assessed using validated instruments such as Endometriosis Health Profile 30 (EHP-30), Short Form Health Survey-36 (SF-36), Visual Analog Scale (VAS) for pain, and related symptoms affecting the QoL	Outcomes not related to the QoL or irrelevant to the research question
Language	Published in English	Published in languages other than English
Study period	Studies published up to the date of data extraction (May 20, 2024)	Studies published after the date of data extraction (May 20, 2024)

Data Collection and Analysis

Articles were gathered from the five specified databases, and the study selection criteria were applied to identify eligible studies. The articles were categorized based on study design, focusing on randomized controlled trials (RCTs) and observational studies while excluding case reports, reviews, and editorials. Information collected from each article included the title, author names, publication date, and details pertinent to the research aim.

Quality assessment tools relevant to the study design were utilized to evaluate each article for potential bias. After assessing the risk of bias, the accepted articles were grouped by key topics related to the impact of interventions, such as exercise, acupuncture, and surgical treatments, on the quality of life in women with endometriosis. These topics are discussed in detail in the Results section.

Results

**Figure 1:**
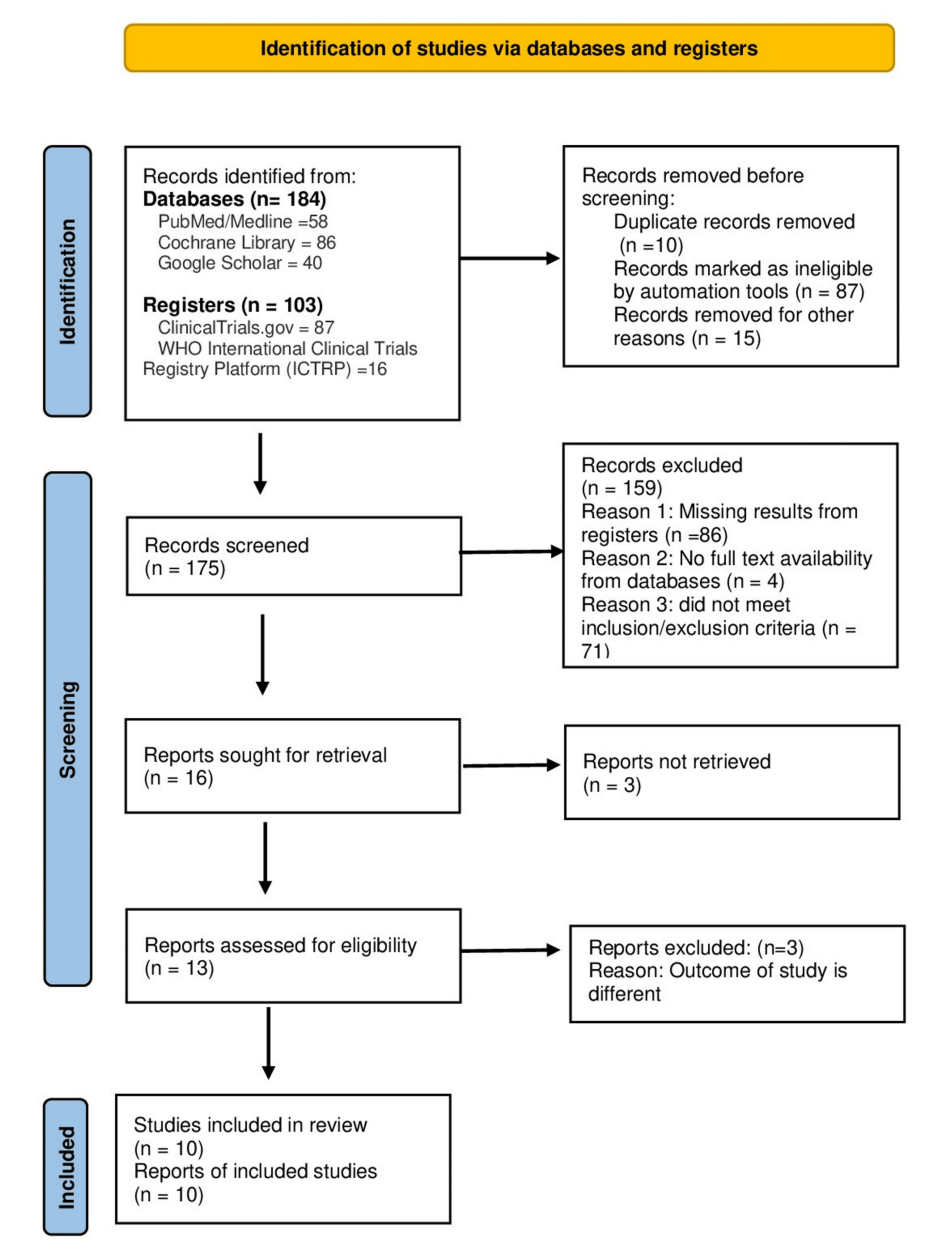
PRISMA flow diagram PRISMA: Preferred Reporting Items for Systematic Reviews and Meta-Analyses

The PRISMA flow diagram in Figure 1 outlines the screening process, including 10 studies. The study selection process involved several steps. First, duplicate and ineligible records were removed, leaving 175 records for screening. During the screening stage, a total of 159 records were excluded. The reasons for exclusion were as follows: 86 records were missing results from registers, four records did not have full-text availability from databases, and 71 records did not meet the inclusion/exclusion criteria.

After the screening process, 16 reports were identified for retrieval, but three of these were not successfully obtained. Upon assessing the remaining 13 reports, an additional three were excluded due to differing study outcomes.

The final review included a total of 10 studies. This comprised six randomized controlled trials, three prospective observational studies, and one retrospective study. Figure 1 highlights these details.

Risk of Bias Assessment

Quality assessment was conducted for all included studies using appropriate tools tailored to their study designs. The Cochrane Risk of Bias Tool was applied to evaluate six randomized controlled trials (RCTs) [18], while the Newcastle-Ottawa Scale (NOS) was utilized for three prospective observational studies and one retrospective study [19]. These assessments ensured a rigorous evaluation of study quality. The NOS criteria focused on three main aspects: the selection of study groups, comparability between study and control groups, and the assessment of study outcomes. Each study was rated on a scale from 0 to 9 points. Studies achieving a score of at least 7 out of 9 (equivalent to 77%) were deemed acceptable for inclusion in the review. The lack of a control group in observational studies leads to selection and measurement bias. Table 3 summarizes the quality assessment results for observational studies.

**Table 3:** Quality assessment results for observational studies -, no score awarded; *, one point out of nine for quality assessment; **, two points awarded

Study	Selection				Comparability	Outcome			Total
Criteria	Representativeness of exposed cohort	Selection of nonexposed group	Ascertainment of exposure	Outcome of interest was not present at the start	Comparability of groups based on design and analysis	Assessment of outcome	Was follow-up long enough?	Adequacy of follow-up	Score out of 9
Ekine et al. (2020) [20]	*	-	*	*	*	*	*	*	7
Pontis et al. (2016) [21]	*	-	*	*	*	*	*	*	7
Lyons et al. (2006) [22]	*	-	*	*	**	*	*	*	8
Legendri et al. (2022) [23]	*	-	*	*	*	*	*	*	7

All six included randomized controlled trials (RCTs) underwent thorough quality assessment using the Cochrane Risk of Bias Tool, which evaluates five critical domains: bias arising from the randomization process, bias due to deviations from the intended intervention, bias due to missing outcome data, bias in the measurement of the outcome, and bias in the selection of the reported result. Each domain was assessed for risk as low (+), some concerns (-), or high risk (*). To ensure rigorousness, only studies with a low risk of bias in at least two out of five domains were included in the systematic review. Most of the bias in the randomized controlled trials was due to insufficient blinding and bias in the measurement of outcome. Appropriate adjustments were made for confounding variables, thus enhancing the quality of these studies. The summary of the quality assessment using the Cochrane Risk of Bias Tool can be seen in Table 4.

**Table 4:** Quality assessment results for randomized controlled trials Domain 1, bias arising from the randomization process; Domain 2, bias due to deviations from the intended intervention; Domain 3, bias due to missing outcome data; Domain 4, bias in the measurement of the outcome; Domain 5, bias in the selection of the reported result. +, low risk; -, some concerns

	Domain 1	Domain 2	Domain 3	Domain 4	Domain 5	Overall
Abbott et al. (2004) [24]	+	+	+	+	+	+
Gallicchio et al. (2015) [25]	+	+	+	+	+	+
Roman et al. (2018) [26]	+	-	+	-	+	+
Li et al. (2023) [27]	+	+	-	-	-	-
de Sousa et al. (2016) [28]	+	+	+	+	+	+
Artacho-Cordón et al. (2023) [29]	+	-	+	+	+	+

Characteristics of the Included Studies

The characteristics of the 10 studies included in this review are presented in Table 5. These studies employed a variety of designs, with six studies being randomized controlled trials (RCTs), three prospective observational studies, and one retrospective study. We divided the table based on the study design, target group, sample size, intervention, outcome measurement, and results. Sample sizes ranged from a minimum of seven to a maximum of 148 participants, including a total of 590 participants. The studies consistently demonstrate significant improvements in pain and the quality of life (QoL) across different interventions with p-scores typically less than 0.001, indicating robust statistical significance. Surgical interventions, such as laparoscopic and transurethral surgeries, bowel resection, and minimally invasive procedures, were conducted. Acupuncture interventions targeted pain reduction and emotional well-being, while exercise interventions focused on improving the QoL. Pain and the QoL were assessed using various scales such as the Visual Analog Scale (VAS), Numerical Rating Scale (NRS-11), Endometriosis Health Profile (EHP-5 and EHP-30), Short Form Health Survey (SF-12 and SF-36), EuroQol-5 Dimension (EQ-5D), Female Sexual Function Index (FSFI), and Beck Depression Inventory (BDI).

**Table 5:** Summary of included studies EHP-36, Endometriosis Health Profile 36; VAS, Visual Analog Scale; NRS-11, Numerical Rating Scale 11; FSFI, Female Sexual Function Index; SF-36, Short Form Health Survey 36; EQ-5D, EuroQol-5 Dimension; MPI, Multidimensional Pain Inventory; BDI, Beck Depression Inventory; POMS, Profile of Mood States; KESS, Knowles-Eccersley-Scott Symptom; GIQLI, Gastrointestinal Quality of Life Index; USP, Urinary Symptom Profile

Study details	Study design	Target group	Control group	Sample size	Intervention	Outcome measurement	Outcome	Results
Ekine et al. (2020) [20]	Cross-sectional prospective study	Women aged 18-45 with invasive endometriosis	No control group	87	Laparoscopic surgical procedures	EHP-36, VAS, and NRS-11	Postoperative quality of life (QoL) and general well-being	1) NRS-11 pain score dropped from 94.8% moderate/severe pain to 18.4% mild pain (p < 0.001). 2) VAS pain score decreased from 8 ± 2.11 to 0.47 ± 1.24 (p < 0.001). 3) Pre-surgery, 89.6% rated the QoL as very bad; post-surgery, 93.1% rated it as significantly improved. 4) General well-being was very low in 93.1% pre-surgery and rated very well or good by 94.2% post-surgery. 5) Of the women with infertility, 58.45% became pregnant, with a 77.4% live birth rate. 6) Sexual life is rated good or very good by 77.85% and 74.7% of women post-surgery (p < 0.001)
Pontis et al. (2016) [21]	Prospective observational study	Women with symptomatic bladder endometriosis	No control group	16	Transurethral + laparoscopic surgical procedure	VAS, SF-36, and FSFI	Postoperative improvement in the QoL and sexual function	1) Statistically significant reduction in pain at one, six, and 12 months post-surgery. 2) Quality of life (SF-36): physical function, significant improvement (p < 0.01); general health, significant improvement (p < 0.00021); physical roles, significant improvement (p < 0.0003); emotional roles, significant improvement (p < 0.03); mental health, significant improvement (p < 0.004); and vitality, significant improvement (p < 0.0013). 3) Sexual function: significant improvement in all six domains and total score at 12 months post-surgery
Lyons et al. (2006) [22]	Prospective observational cohort study	Women with bowel endometriosis	No control group	7	Laparoscopic endometrial bowel resection	EQ-5D, SF-12, and Sexual Activity Questionnaire	Postoperative improvement in the QoL and menstrual pain	1) Pain reduction: dysmenorrhea, 71-5 (p = 0.028); non-menstrual pelvic pain, 74-11 (p = 0.046); dyspareunia, 66-5 (p = 0.080); and dyschezia, 48-20 (p = 0.173). 2) Quality of life: improved in all measures (SF-12, EuroQoL, and Sexual Activity Questionnaire) at 12 months. 3) Fertility: all three women wishing to conceive succeeded, resulting in three live births
Legendri et al. (2022) [23]	Retrospective study	Women who underwent minimally invasive surgery for painful deep infiltrating endometriosis (DIE)	No control group	54	Minimally invasive surgery	EHP-5 and VAS	Postoperative improvement in the QoL and pain	EHP-5 scores: pre-operation 61.36 (42.18-68.75) versus two-year post-operation 20.45 (0-38.06), p < 0.001; VAS for dysmenorrhea: pre-operation 8 (7-9.75) versus two-year post-operation 3 (2-5.25), p < 0.001; VAS for dyspareunia: pre-operation 6 (3.1-8.9) versus two-year post-operation 3 (0-6), p < 0.001
Abbott et al. (2004) [24]	Randomized, blinded, crossover study	Women with proven endometriosis who underwent laparoscopic surgery	Diagnostic surgery group	39	Laparoscopic surgery	VAS, EQ-5D, SF-12, and Sexual Activity Questionnaire	Effect on pain and the QoL after laparoscopic surgery versus placebo surgery	Symptomatic improvement: excisional surgery, 80% (16/20); placebo, 32% (6/19); statistical significance, χ²(1) = 9.3; and quality of life, significant improvement six months after excisional surgery, not after placebo
Gallicchio et al. (2015) [25]	Randomized controlled trial	Women undergoing surgery for endometriosis and white light (WL) imaging followed by narrow-band imaging (NBI) was used	Women undergoing surgery for endometriosis and only white light imaging used	148	Laparoscopic surgery with narrow-band imaging and white light imaging versus only white band imaging	VAS and EHP-30	Effect on pain reduction and the QoL after laparoscopic surgery	Pain reduction: both the WL/NBI and WL/WL groups showed similar reductions in pain at three and six months post-surgery. QoL improvement: both groups experienced significant improvements in QoL at three and six months, with no significant difference between the two groups. Statistical data: the sensitivity to detect endometriotic lesions was significantly higher using NBI (100%) compared to white light alone (78.8%, p < 0.001)​
Roman et al. (2018) [26]	Randomized controlled trial	Women undergoing radical rectal surgery for large deep infiltrating endometriosis involving the rectum	Women undergoing conservative surgery for large deep infiltrating endometriosis involving the rectum	60	Radical rectal surgery versus conservative surgery for endometriosis	VAS, KESS, GIQLI, Wexner, USP, and SF-36	Functional outcome after surgery	Functional symptoms 24 months post-surgery: conservative surgery, 48.1% (13 patients); segmental resection, 39.4% (13 patients); odds ratio, 0.70 (95% CI: 0.22-2.21; p = 0.70); and KESS, GIQLI, Wexner, USP, SF-36, and VAS scores, similar in both groups
Li et al. (2023) [27]	Multicenter, randomized, single-blind, placebo-controlled trial	Women with endometriosis-associated pain aged between 20 and 40 years who received acupuncture	Women with endometriosis-associated pain aged between 20 and 40 years who received sham acupuncture	106	Acupuncture versus sham acupuncture	VAS, MPI, BDI, POMS, and EHP-12	Pain reduction, the quality of life, and emotional state	Pain reduction: dysmenorrhea, VAS score decreased by 3.9 points in the acupuncture group (p < 0.001); non-menstrual pelvic pain, no significant difference; dyspareunia, no significant difference; quality of life and emotional state, significant improvements in MPI, BDI, POMS, and EHP scores at week 12 (p < 0.001); and no significant differences at week 24
de Sousa et al. (2016) [28]	Randomized controlled trial	Women with endometriosis undergoing acupuncture treatment	Women with acupuncture undergoing placebo therapy	42	Acupuncture versus placebo therapy	VAS and EHP-30	Effect on chronic pelvic pain (CPP) and the QoL	Chronic pelvic pain (CPP) and dyspareunia: improvement, significant improvement in both groups (p < 0.0001); CPP reduction, 66% in the the treatment group versus 17% in the placebo group; dyspareunia reduction, 65% in the the treatment group versus 13% in the placebo group; quality of life (QoL), no significant QoL improvement in the placebo group despite pain reduction
Artacho-Cordón et al. (2023) [29]	Randomized controlled trial	Women with endometriosis symptoms enrolled in Physio-EndEA	Women with endometriosis with routine management	31	Supervised exercise	EHP-30	QoL pain intensity	QoL, improved post-intervention and at one-year follow-up with large effect sizes (d > 0.80); pain, reduced dyspareunia and catastrophic thoughts; strength and stability, increased abdominal and back strength and lumbopelvic stability; muscle architecture, increased thickness of transversus abdominis and width of lumbar multifidus

Discussion

This systematic review, conducted following the Preferred Reporting Items for Systematic Reviews and Meta-Analyses (PRISMA) guidelines, aimed to investigate the effectiveness of surgery, acupuncture, and exercise programs in improving symptoms and the quality of life in women with endometriosis. A comprehensive search strategy was employed to identify and include relevant studies (n = 10) for synthesis.

The systematic review revealed that several of the included studies demonstrated promising results. The participants who underwent surgical interventions, received acupuncture treatments, or engaged in exercise programs exhibited significant improvements in pain scores and quality of life measures compared to control groups. These positive outcomes were observed across the different types of interventions, though the degree of effectiveness varied.

Surgical Intervention

The majority of the included studies focused on surgical interventions, primarily the laparoscopic excision of endometriotic lesions. Consistently, these studies reported significant reductions in pain and improvements in quality of life post-surgery.

The most impactful findings came from Ekine et al. (2020) [20], who reported a significant drop in Numerical Rating Scale (NRS-11) pain score from 94.8% moderate/severe pain to 18.4% mild pain (p < 0.001). The Visual Analog Scale (VAS) pain score also decreased from 8 ± 2.11 to 0.47 ± 1.24 (p < 0.001). Additionally, 93.1% of the patients rated their quality of life as significantly improved post-surgery.

Similarly, Pontis et al. (2016) [21] found statistically significant reductions in pain and improvements in multiple SF-36 quality-of-life domains and sexual function at 12 months post-surgery. Lyons et al. (2006) [22] reported significant decreases in dysmenorrhea and non-menstrual pelvic pain, along with improvements in all quality-of-life measures at 12 months.

Legendri et al. (2022) [23] observed significant improvements in Endometriosis Health Profile (EHP-5) and VAS scores for dysmenorrhea and dyspareunia at two years post-surgery. Abbott et al. (2004) [24] demonstrated significant symptomatic improvement with excisional surgery compared to placebo, with 80% of the excisional surgery group showing improvement versus 32% in the placebo group.

Gallicchio et al. (2015) [25] found similar pain reduction and quality-of-life improvements in both narrow-band imaging and white light imaging groups at three and six months post-surgery. Roman et al. (2018) [26] reported no significant difference in functional outcomes between radical and conservative surgery groups at 24 months post-surgery.

The recurrence of symptoms in some patients suggests that while surgery is effective in the short term, it may not provide a permanent solution for all individuals. Factors such as the extent of endometriosis, the precision of the surgical technique, and postoperative care could influence these outcomes. Future studies should focus on long-term follow-up and strategies to prevent recurrence, such as combining surgery with other therapeutic approaches.

Acupuncture Intervention

Two studies on acupuncture for endometriosis-related pain have both demonstrated significant reductions in pain and improvements in overall well-being.

Li et al. (2023) [27] reported a significant decrease in the Visual Analog Scale (VAS) score for dysmenorrhea by 3.9 points in the acupuncture group, compared to the control group (p < 0.001). Additionally, the acupuncture group showed significant improvements in Multidimensional Pain Inventory (MPI), Beck Depression Inventory (BDI), Profile of Mood States (POMS), and Endometriosis Health Profile (EHP) scores at week 12 (p < 0.001).

Similarly, de Sousa et al. (2016) [28] found significant improvement in chronic pelvic pain and dyspareunia in both groups, with a 66% reduction in chronic pelvic pain and 65% reduction in dyspareunia in the acupuncture treatment group, compared to the control group (p < 0.0001).

The noninvasive nature of acupuncture and its potential to alleviate pain without the side effects associated with pharmacological treatments make it an appealing option for many women with endometriosis. However, variations in outcomes may be attributed to differences in acupuncture techniques, session frequency, and duration. Further research with standardized protocols and larger sample sizes is necessary to validate these findings and optimize treatment regimens. Additionally, exploring the mechanisms by which acupuncture affects endometriosis-related pain could provide valuable insights into its therapeutic potential.

Exercise Intervention

The single study on exercise programs included in this review reported notable improvements in pain and physical function.

Artacho-Cordón et al. (2023) [29] showed large effect size improvements (d > 0.80) in the quality of life post-intervention and at one-year follow-up. The exercise program led to decreased dyspareunia and catastrophic thoughts related to pain. Additionally, the participants experienced increased abdominal and back strength, improved lumbopelvic stability, and enhanced muscle architecture.

Exercise is known to enhance overall physical health, reduce stress, and improve mood, which may collectively contribute to the observed benefits. However, the mixed results, with some participants experiencing minimal improvements or slight worsening of symptoms, underscore the need for individualized exercise plans. Factors such as the intensity, duration, and type of exercise, as well as the baseline fitness level of the participants, are crucial considerations.

Future research should aim to establish evidence-based guidelines for exercise programs tailored to women with endometriosis. This would involve exploring the optimal exercise prescription, accounting for individual variations in disease severity, fitness level, and treatment goals. Investigating the mechanisms by which exercise mediates improvements in endometriosis-related symptoms could also provide valuable insights to improve therapeutic outcomes.

Comparison with existing evidence

Our findings are consistent with the conclusions of previous systematic reviews and meta-analyses on the management of endometriosis-related symptoms and quality of life.

For surgical interventions, reviews by Bafort et al. (2020) [30] and Duffy et al. (2014) [31] have reported significant improvements in pain and the quality of life following the laparoscopic excision of endometriotic lesions, corroborating the positive surgical outcomes in our review.

Regarding acupuncture, a systematic review by Giese et al. (2023) [32] has demonstrated acupuncture's effectiveness in reducing pain and improving the quality of life in women with endometriosis, in line with the benefits observed in our analysis.

For exercise interventions, a review by Riazi et al. (2015) [33] has found that exercise programs, particularly aerobic and pelvic floor muscle training, can lead to substantial reductions in pain and enhancements in physical function and the quality of life, supporting the positive impacts reported in our review.

By aligning with the conclusions of these previous high-quality syntheses of the evidence, our findings add to the growing body of literature supporting the use of surgical-, acupuncture-, and exercise-based interventions for improving the quality of life and managing the symptoms of endometriosis.

Strengths of the review

A thorough search across multiple databases and grey literature minimized publication bias, providing a comprehensive overview of current evidence on surgical, acupuncture, and exercise interventions for endometriosis. By examining variations in intervention types, intensities, and participant demographics, the review highlights the need for tailored research and identifies gaps in current evidence, paving the way for more effective treatment strategies. Addressing these aspects, the review consolidates existing knowledge and offers valuable insights for future research and clinical practice in managing endometriosis-related chronic pain.

Limitations of included studies and review process

Some included studies were observational, limiting causal inference about the effectiveness of interventions. Ekine et al. (2020) [20] and Pontis et al. (2016) [21] lacked control groups, weakening the conclusions drawn. Some studies had methodological limitations such as small sample sizes, such as the seven participants in Lyons et al. (2006) [22]; the lack of blinding, as seen in de Sousa et al. (2016) [28] and Artacho-Cordón et al. (2023) [29]; and potential selection bias, all of which may affect the reliability of findings. The review focused on English-language studies, possibly excluding relevant research in other languages, leading to an incomplete understanding of global evidence.

Future research directions

The findings of this review suggest that surgical-, acupuncture-, and exercise-based approaches may potentially offer beneficial effects for women with endometriosis. However, the review also highlights the need for further research to better understand the comparative effectiveness, optimal treatment protocols, and long-term impacts of these interventions. The continued exploration of these management strategies could help inform clinical decision-making and enhance the comprehensive care of individuals affected by this complex and debilitating condition.

To improve the understanding of treatments for endometriosis, future research should prioritize several key areas. Primarily, conducting well-designed, adequately powered randomized controlled trials is essential to establish causal relationships and compare the effectiveness of different surgical techniques, acupuncture protocols, or exercise programs. These trials should aim to elucidate the comparative efficacy of the various interventions and explore whether certain subgroups of endometriosis patients, based on factors such as age, baseline pain severity, and endometriosis phenotypes, may benefit more from specific treatment approaches.

Additionally, a focus on the long-term adherence and sustainability of interventions, particularly for exercise programs, is necessary to ensure lasting improvements in symptoms and the quality of life. Longitudinal studies tracking the persistence of treatment effects over extended follow-up periods will provide valuable insights into the durability of the observed benefits.

By addressing these critical gaps in the existing literature, future research can contribute to the development of more effective, personalized treatment strategies for managing endometriosis. This, in turn, will enhance comprehensive care and ultimately improve the quality of life for women affected by this debilitating condition.

## Conclusions

This comprehensive systematic review synthesizes the current evidence on the effectiveness of various interventions, including surgery, acupuncture, and exercise programs, for improving the quality of life in women with endometriosis. The findings indicate that these approaches can provide significant benefits for this patient population. The majority of the included studies demonstrated that surgical interventions, particularly the laparoscopic excision of endometriotic lesions, led to considerable reductions in pain and improvements in the quality of life. Acupuncture also showed promising results, with studies reporting meaningful decreases in pain scores and enhancements in overall well-being. Furthermore, the single exercise program evaluated in this review highlighted substantial improvements in the quality of life, physical function, and pain reduction. However, the review also underscores the need for more personalized and tailored approaches, as some participants experienced limited or mixed results with the interventions. Despite the limitations of the current evidence, this systematic review consolidates the existing knowledge and provides valuable insights for clinicians and researchers working to improve the quality of life for individuals affected by this chronic and debilitating condition.
